# Genomic Epidemiology of Salmonella enterica Circulating in Surface Waters Used in Agriculture and Aquaculture in Central Mexico

**DOI:** 10.1128/aem.02149-21

**Published:** 2022-03-08

**Authors:** N. E. Ballesteros-Nova, S. Sánchez, J. L. Steffani, L. C. Sierra, Z. Chen, F. A. Ruíz-López, R. L. Bell, E. A. Reed, M. Balkey, M. S. Rubio-Lozano, O. Soberanis-Ramos, F. Barona-Gómez, E. W. Brown, M. W. Allard, J. Meng, E. J. Delgado-Suárez

**Affiliations:** a Facultad de Medicina Veterinaria y Zootecnia, Universidad Nacional Autónoma de México, Mexico City, México; b Evolution of Metabolic Diversity Laboratory, CINVESTAV-IPN, Irapuato, Mexico; c Joint Institute for Food Safety & Applied Nutrition – Food and Drug Administration Center of Excellence and Center for Food Safety & Security Systems (CFS3), University of Maryland, College Park, Maryland, USA; d Office of Regulatory Science, Center for Food Safety and Applied Nutrition, U. S. Food and Drug Administration, College Park, Maryland, USA; e Department of Nutrition and Food Science, University of Maryland, College Park, Maryland, USA; The Pennsylvania State University

**Keywords:** *Salmonella*, surface water, irrigation, aquaculture, whole-genome sequencing, serotyping, transmission dynamics, phylogenetic analysis, Mexico

## Abstract

Salmonella enterica can survive in surface waters (SuWa), and the role of nonhost environments in its transmission has acquired increasing relevance. In this study, we conducted comparative genomic analyses of 172 S. enterica isolates collected from SuWa across 3 months in six states of central Mexico during 2019. S. enterica transmission dynamics were assessed using 87 experimental and 112 public isolates from Mexico collected during 2002 through 2019. We also studied genetic relatedness between SuWa isolates and human clinical strains collected in North America during 2005 through 2020. Among experimental isolates, we identified 41 S. enterica serovars and 56 multilocus sequence types (STs). Predominant serovars were Senftenberg (*n* = 13), Meleagridis, Agona, and Newport (*n* = 12 each), Give (*n* = 10), Anatum (*n* = 8), Adelaide (*n* = 7), and Infantis, Mbandaka, Ohio, and Typhimurium (*n* = 6 each). We observed a high genetic diversity in the sample under study, as well as clonal dissemination of strains across distant regions. Some of these strains are epidemiologically important (ST14, ST45, ST118, ST132, ST198, and ST213) and were genotypically close to those involved in clinical cases in North America. Transmission network analysis suggests that SuWa are a relevant source of S. enterica (0.7 source/hub ratio) and contribute to its dissemination as isolates from varied sources and clinical cases have SuWa isolates as common ancestors. Overall, the study shows that SuWa act as reservoirs of various S. enterica serovars of public health significance. Further research is needed to better understand the mechanisms involved in SuWa contamination by S. enterica, as well as to develop interventions to contain its dissemination in food production settings.

**IMPORTANCE** Surface waters are heavily used in food production worldwide. Several human pathogens can survive in these waters for long periods and disseminate to food production environments, contaminating our food supply. One of these pathogens is Salmonella enterica, a leading cause of foodborne infections, hospitalizations, and deaths in many countries. This research demonstrates the role of surface waters as a vehicle for the transmission of Salmonella along food production chains. It also shows that some strains circulating in surface waters are very similar to those implicated in human infections and harbor genes that confer resistance to multiple antibiotics, posing a risk to public health. This study contributes to expand our current knowledge on the ecology and epidemiology of Salmonella in surface waters.

## INTRODUCTION

Nontyphoidal Salmonella (NTS) is one of the most common causes of foodborne diseases in the world (1). It is commonly associated with foods of animal origin (i.e., meat, poultry, eggs, seafood) and fresh produce (2). However, SuWa have been identified as a vehicle for the introduction of the pathogen into the food chain (3, 4). An increasing number of salmonellosis outbreaks have been linked to SuWa used for the irrigation of fruits and vegetables that are consumed raw, such as tomatoes, cantaloupes, sprouts, green leafy vegetables, and berries, among others (5–11).

Numerous studies have shown NTS survives for up to a month in SuWa (9, 12). However, it may persist for longer periods by forming biofilms or due to continuous reintroduction events from natural reservoirs such as protozoa, vertebrates, and surface runoff (3). Hence, it is important to monitor NTS contamination of SuWa used for crop irrigation, animal production, or aquaculture. This is especially important in countries like Mexico, which is one of the biggest exporters of fresh produce in the world (13), as well as a heavy producer of meat, poultry, and aquaculture products (14).

There are few Mexican studies on NTS SuWa contamination, most of them conducted in the Culiacán Valley (Northwest region), which is characterized by intensive crop and aquaculture production (15). For instance, a 39% NTS prevalence was reported in irrigation canals from the Culiacán Valley in 2009 (16), with a strong predominance (65%) of isolates of serovar Typhimurium exhibiting tetracycline resistance phenotypes. Moreover, studies in rivers of the same region reported more than 80% NTS prevalence, with a high serovar and genetic diversity (17), and more than 40% of these isolates showed multidrug-resistant phenotypes (4). Conversely, little is known about NTS populations in SuWa from other regions of Mexico that are also used in food production.

Currently, whole-genome sequencing (WGS) is recognized as a robust method to characterize organisms. Its increasing use worldwide has allowed attaining significant progress in our understanding of the epidemiology of infectious diseases, including foodborne salmonellosis. For that reason, this research adopted a genomic surveillance approach to study the serovar and genetic diversity of NTS circulating in SuWa across six states from central Mexico.

## RESULTS

### Serovar diversity and AMR genotypes.

We identified 41 serovars among the 172 Salmonella strains analyzed (Table 1). The predominant serovars were Senftenberg, Meleagridis, Agona, Newport, Give, Anatum, Adelaide, Infantis, Mbandaka, Ohio, and Typhimurium. Collectively, these serovars represented nearly 60% of the strains being studied (99 of 172), with most of them scattered across water sources from three or four Mexican states. The prevalence of Salmonella serovars was not associated with the type of SuWa body (χ^2^ = 30.6, *P* = 0.7220), water temperature (χ^2^ = 14.1, *P* = 0.1181), or turbidity (χ^2^ = 19.1, *P* = 0.3875). However, water pH did influence the relative serovar representation (χ^2^ = 40.0, *P* = 0.0021), with over 70% of all serovars isolated from alkaline waters (pH 7.2 to 9.3).

**TABLE 1:** Distribution of major Salmonella serovars isolated from SuWa across the studied regions

Serovar	No. of isolates^*a*^						Total
	GTO	HGO	CDMX	MEX	MOR	TLAX	
Senftenberg	7		2	3	2		14
Newport	7	1		1	3		12
Meleagridis	7		3		1	1	12
Agona	7			1	1	3	12
Give	3			2	4	1	10
Anatum	4			2	1	1	8
Adelaide		1		3	3		7
Infantis	3			2		1	6
Mbandaka	3	1	1	1			6
Ohio	6						6
Typhimurium	3	2		1			6
Others	29	5	6	15	18		73
Total	79	10	12	31	33	7	172

a Mexican states: GTO, Guanajuato; HGO, Hidalgo; CDMX, Mexico City; MOR, Morelos; TLAX, Tlaxcala.

**FIG 1:**
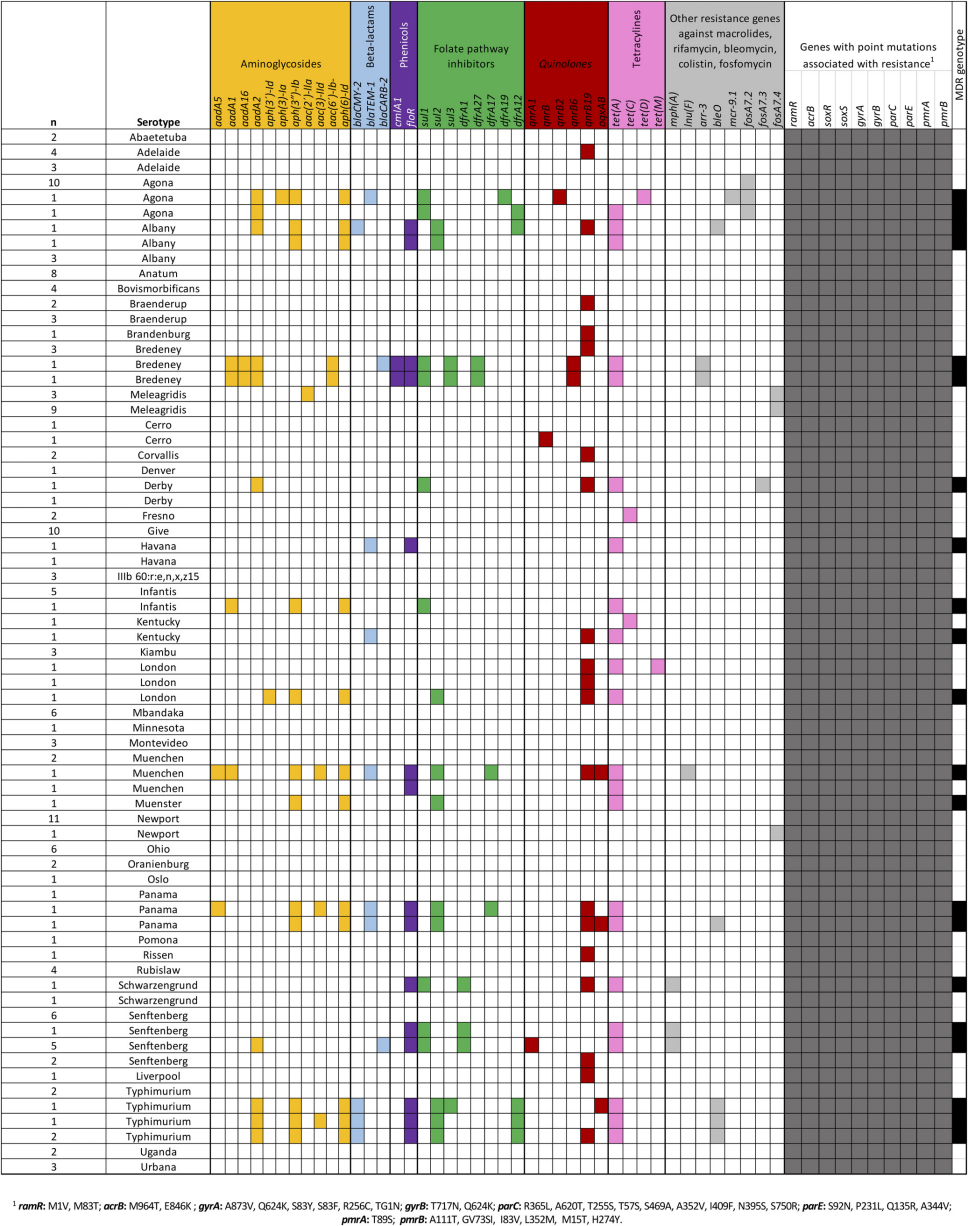
Genotypic antimicrobial resistance (AMR) profile of 172 Salmonella isolates from surface waters (SuWa). Antibiotic classes are color-coded, and cells filled with the corresponding antibiotic class color indicate that the AMR gene is present. The rightmost columns report the presence of point mutations in the listed genes (dark gray cells) and the occurrence of multidrug-resistant (MDR) genotypes (black cells). Blank cells indicate the absence of AMR genes, mutations, or MDR genotypes. The results are summarized considering the number of isolates of the same serovar with the same AMR profile. Individual results and isolate metadata are provided in Table S1 in and Fig. 2.

There was also an association between Salmonella serovar and antimicrobial resistance (AMR) genotypes (χ^2^ = 47.0, *P* = 0.0004). Genotypic multidrug-resistant (MDR) profiles (26 of 172) were more frequently found in isolates of serovars Typhimurium (4 of 6), Senftenberg (6 of 12), Panama (2 of 3), Bredeney (2 of 3), and Albany (2 of 5) (Fig. 1). Some of these isolates carried AMR genes against six or more antimicrobial classes. Conversely, isolates of other serovars (120 of 172) were not predicted to carry any AMR genes (i.e., Abaetetuba, Anatum, Bovismorbificans, Denver, Kiambu, Mbandaka, Minnesota, Montevideo, Newport, Ohio, among others). However, all experimental isolates had mutations that are associated with resistance to quinolones (*gyrAB* and *parCE* genes), colistin (i.e., *pmrAB* genes), and macrolides (*acrB* genes) and with MDR phenotypes (*ramR* and *soxRS* genes) (18–22).

### Genetic diversity among newly sequenced Salmonella isolates.

Multilocus sequence typing (MLST) identified 56 different sequence types (STs) among the 172 isolates. Generally, isolates of the same serovar belonged to the same ST, except those exhibiting a polyphyletic behavior, such as Newport (ST118, ST7815, ST45, ST463, and ST132), Give (ST524, ST516, ST654, and ST2589), Typhimurium (ST118, ST2072, and ST213), and Muenchen (ST83, ST112, and ST2881). Strains of ST213 formed a clonal complex (eBURST group) involving most serovar Typhimurium isolates (5 of 6) and were designated founders of other STs of public health significance, such as ST45, ST118, ST132, and ST198 (Fig. S2).

**FIG 2:**
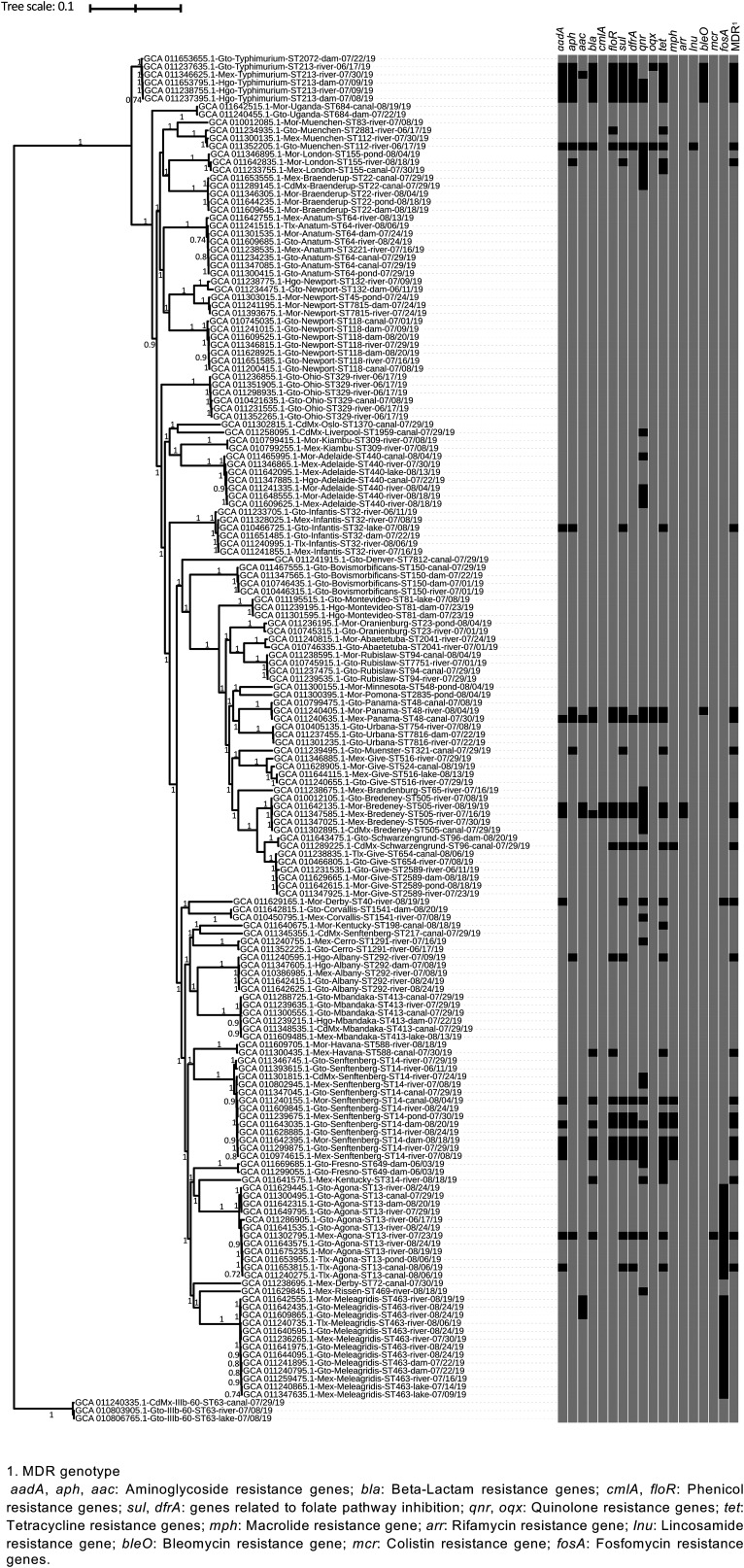
ML tree based on single-nucleotide polymorphism (SNP) analysis of 172 Salmonella isolates from SuWa. Tip labels show NCBI assembly accessions, Mexican state, serovar, sequence types (STs), and type of SuWa body. AMR genotypes are mapped onto the tree. Black cells correspond to isolates that carried at least one variant of the indicated AMR alleles, while gray cells indicate absence of that gene. Clade support is indicated in the branches as bootstrap values, unless it is less than 0.7.

The single-nucleotide polymorphism (SNP) phylogeny was consistent with the results of MLST typing. It revealed a high genetic diversity and a widespread distribution of clonal strains (98 to 100% bootstrap support) across Mexican states (Fig. 2). Experimental isolates were divided into two genetically divergent clades with 100% bootstrap support. There was one small sublineage composed of S. enterica subsp. *diarizonae* isolates (*n* = 3). The other clade comprised the remaining strains (*n* = 169) and was further divided into two major subclades. One subclade included strains of serovar Typhimurium (ST118, ST2103, and ST2072), with most of them (5 of 6) exhibiting MDR genotypes. The remaining strains clustered together in a big subclade with 29 SNP clusters. Within this subclade, isolates of the same serovar were grouped in the same SNP cluster, regardless of their MLST profile, except for some serovars showing polyphyletic behavior (i.e., Derby, Give, Newport, Senftenberg). Chi-square tests showed there was no association between SNP clusters and water temperature (χ^2^ = 5.6, *P* = 0.7796), pH (χ^2^ = 22.4, *P* = 0.3200), or turbidity (χ^2^ = 14.6, *P* = 0.6877). However, some serovars, STs, and SNP clusters were limited to specific Mexican states. For instance, isolates of serovars Bovismorbificans (ST150) and Ohio (ST329) formed single SNP clusters and were isolated only in Guanajuato. Likewise, isolates of serovar Derby (ST40) were isolated only in Morelos. Conversely, MDR genotypes were more frequent in certain SNP clusters (χ^2^ = 29.5, *P* = 0.0005), such as those containing isolates of serovars Senftenberg (ST14), Bredeney (ST505), Derby (ST40), Panama (ST48), and Typhimurium (ST213).

Certain Salmonella genotypes, such as those included in serovar Newport and Senftenberg clades, were consistently isolated for 2 to 3 months in Guanajuato (Fig. 2). For instance, Newport isolates were collected in July through August, while Senftenberg isolates were recovered in June through August. Unfortunately, it was not possible to address Salmonella persistence in water bodies. The set of isolates with WGS data available were collected during the first (*n* = 150) and second (*n* = 22) sampling rounds, of the five that were conducted in 2019. This is a limitation of the study that will be dealt with in future contributions, once the whole set of isolates is sequenced.

### Salmonella transmission dynamics.

**FIG 3:**
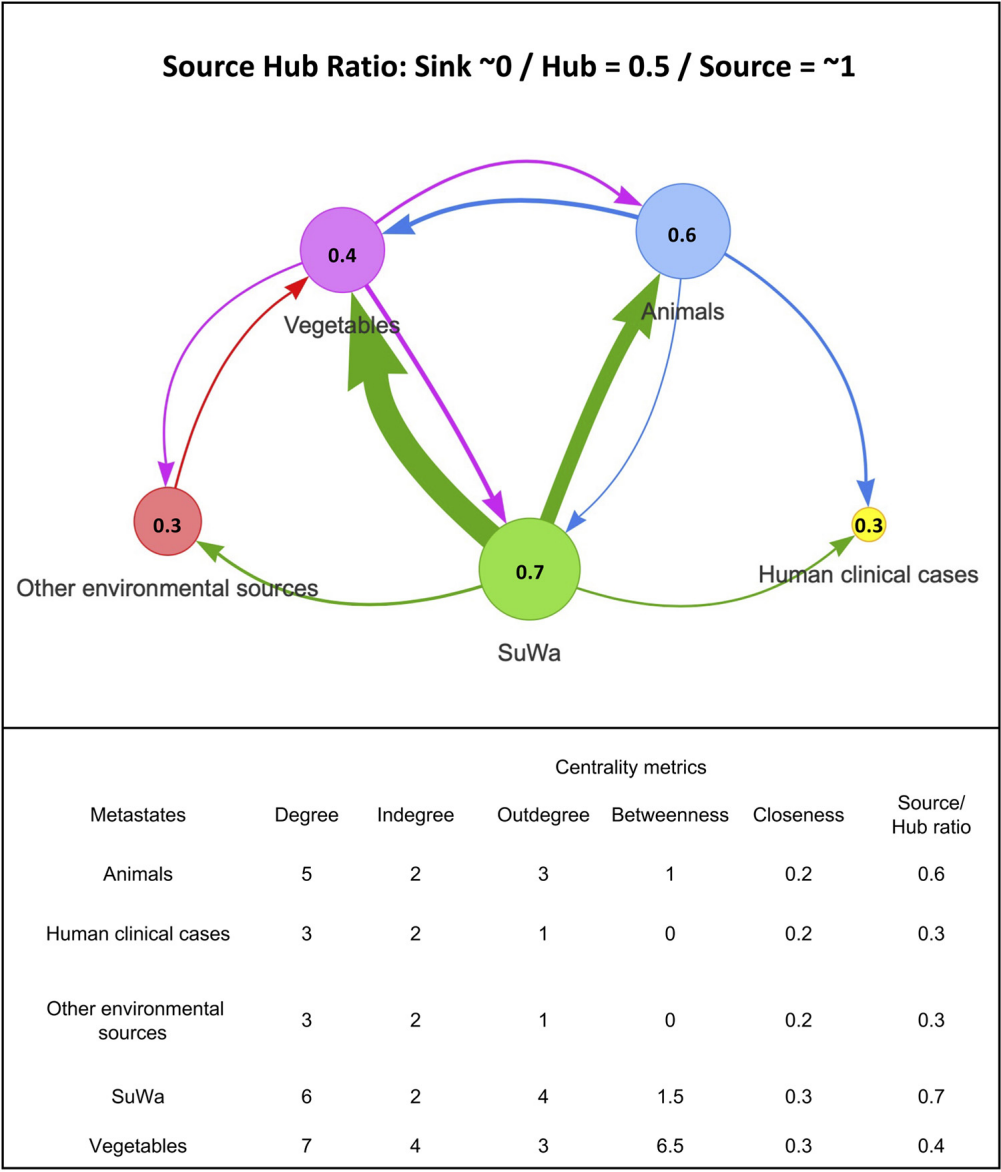
Transmission network of 199 Salmonella isolates collected in Mexico from SuWa (*n* = 105), human clinical cases (*n* = 6), animals (*n* = 28), vegetables (*n* = 55), and the environment (*n* = 5). The network was generated for the source/hub ratio. Each circle corresponds to a specific isolate source, and the source/hub ratio is indicated inside the circle. The values for centrality metrics (degree, indegree, outdegree, betweenness, closeness, and source/hub ratio) are reported below the network. The accession numbers and metadata of isolates used in this analysis are provided in Fig. 4.

**FIG 4:**
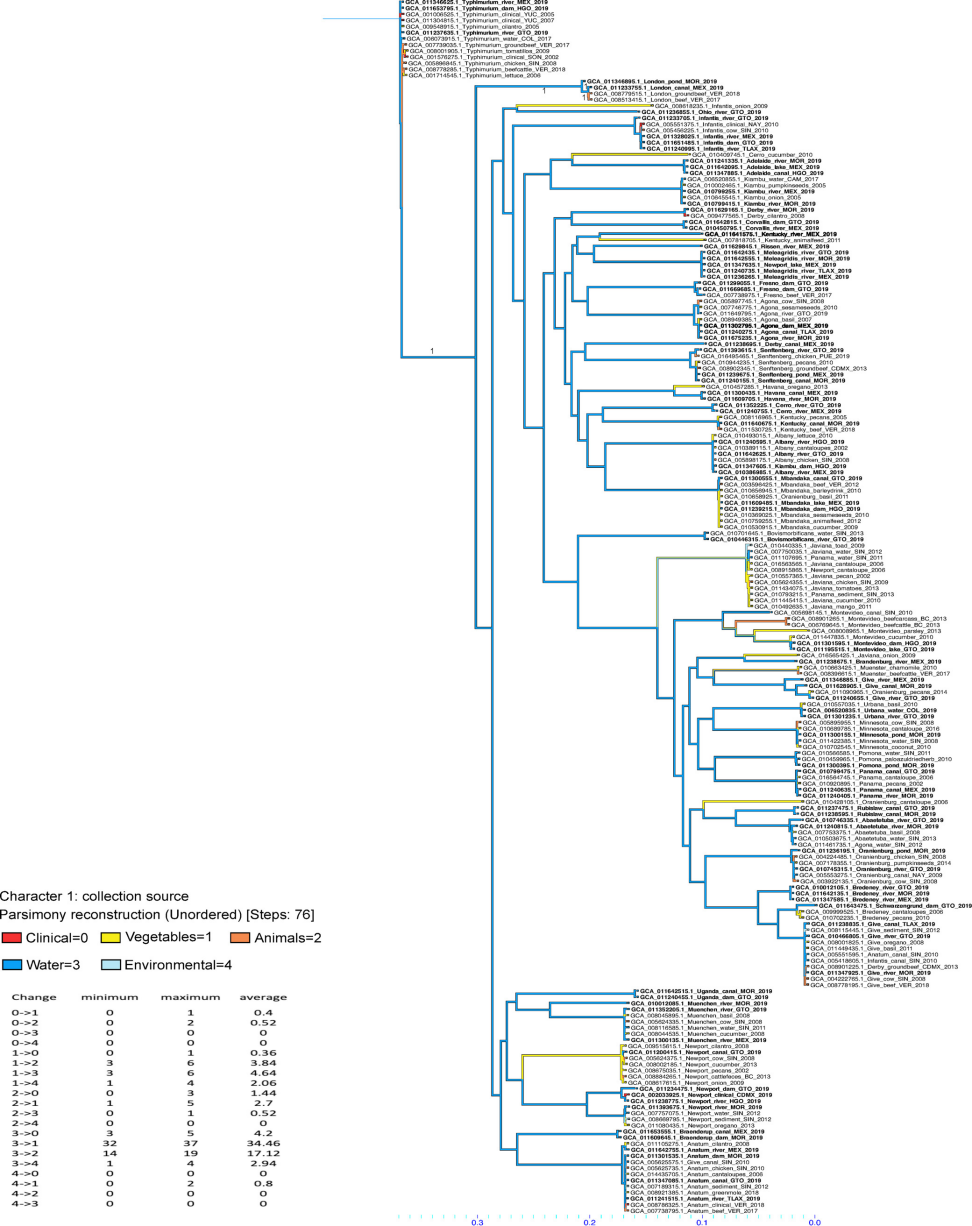
Reconstruction of character states at ancestral nodes in a phylogenetic tree of 199 public Salmonella isolates recovered from multiples sources in Mexico during 2002 through 2019. Isolates from this research are highlighted with bold type. Character states are color-coded according to isolation source, while the summary of changes in character states is summarized next to the tree. NCBI assembly accession, serovar, isolation source, and Mexican state of origin (if available) are indicated at tip labels. BC, Baja California; CAM, Campeche; CDMX, Mexico City; COL, Colima; GTO, Guanajuato; HGO, Hidalgo; MEX: State of Mexico; MOR, Morelos; NAY, Nayarit; SIN, Sinaloa; SON, Sonora; TLAX, Tlaxcala; VER, Veracruz; YUC, Yucatán.

The transmission network showed SuWa is a relevant source of Salmonella (Fig. 3), as it had the highest source/hub ratio (0.7). The thickness of lines and arrows shows the transmission of the pathogen from SuWa predominantly affects vegetables and animals. Nonetheless, transmission patterns were bidirectional, with transitions from SuWa to other sources and vice versa, showing the complexity of this phenomenon. According to betweenness centrality, vegetables, SuWa, and animals were the most important hubs for the traffic of the pathogen (6.5, 1.5, and 1, respectively). However, closeness centrality was mostly equal (0.2 to 0.3) across sources, indicating that every node may act as a direct point of transmission to other nodes. Reconstruction of character states at ancestral nodes with Mesquite (Fig. 4) showed that the ancestors of most clinical isolates originated from SuWa, vegetables, and animals. However, there were a few transitions from clinical cases to vegetables and animals as well.

### Genetic relatedness of isolates from SuWa and clinical strains from North America.

**FIG 5:**
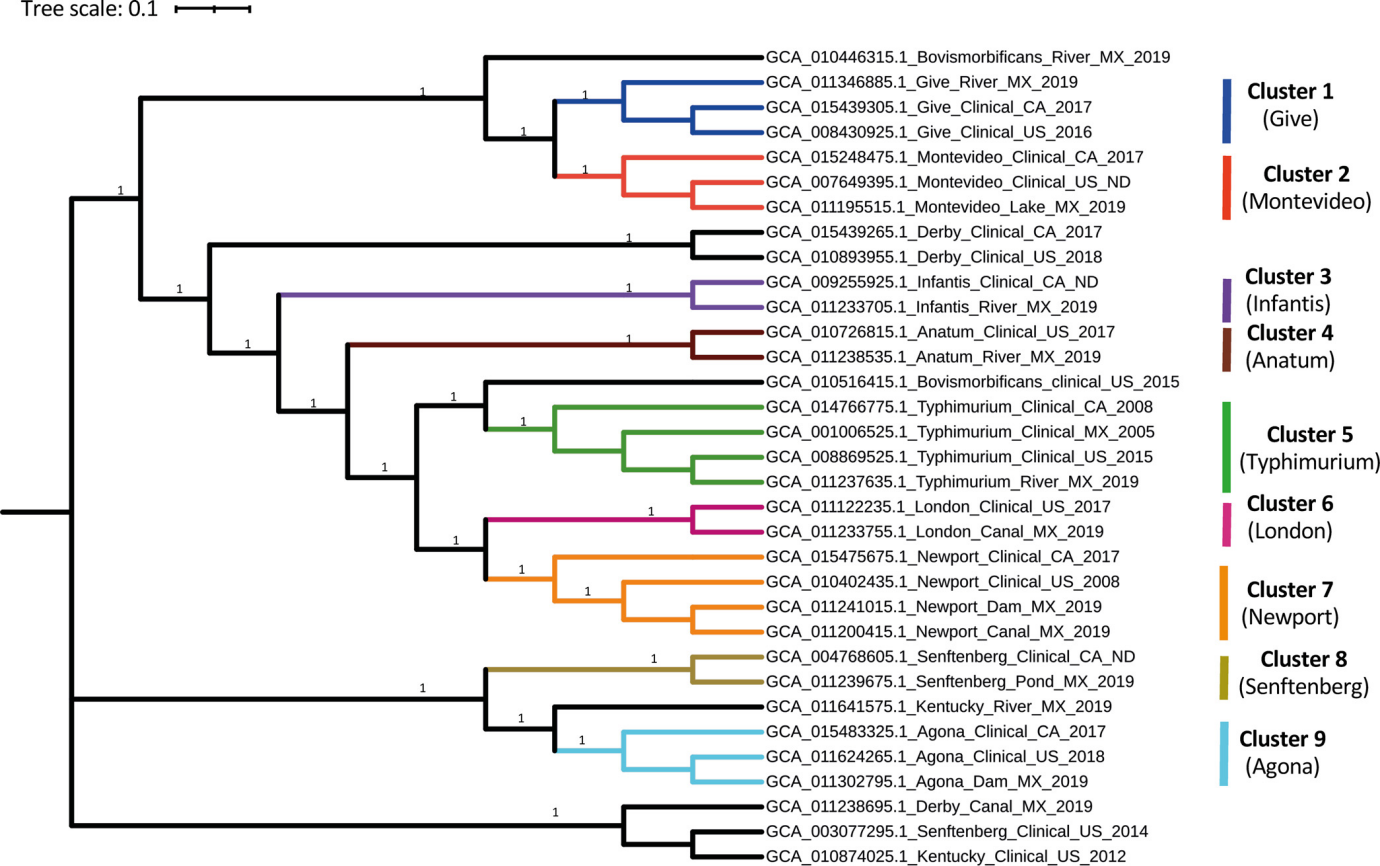
ML tree based on SNP analysis of 33 public Salmonella isolates collected from SuWa in Mexico and from human clinical cases in Mexico (MX), Canada (CA), and the United States (US). Tip labels show NCBI accessions, serovar, isolation source, country of origin, and collection year, unless there was no data (ND) recorded for collection year. Clade support is indicated in the branches as bootstrap values, unless it is less than 0.7. SNP clusters are color-coded and numbered in bold text to facilitate their visualization.

The SNP phylogeny showed isolates from SuWa were closely related (36 to 88 SNP distance) to those involved in human clinical cases in Mexico, the USA, or Canada (Fig. 5). SuWa isolates of serovars Bovismorbificans and Derby, however, were closer to strains of other serovars (i.e., Give and Senftenberg and Kentucky, respectively) than to their clinical counterparts. Additionally, most strains from SuWa in Mexico were more closely related to US clinical strains than those from Canada.

A review of NCBI SNP clusters at the NCBI Pathogen Detection website revealed close to 60% of our experimental SuWa isolates (100 of 172) clustered together with clinical strains from North America. In most of these SNP clusters (80 of 100), the average SNP distance between isolates was less than 21 (Table S1).

## DISCUSSION

In this research, we confirmed that SuWa in the Mexican regions under study were frequently contaminated with a high diversity of S. enterica subsp. *enterica* serovars. These are predominant in relation to strains of other S. enterica subspecies, such as *diarizonae*, that are present in SuWa occasionally. Cold-blooded animals, such as reptiles, are known reservoirs of S. enterica subsp. *diarizonae* strains (23) and disseminate them in the environment. Although human infections caused by subsp. *diarizonae* are rare (24, 25), future studies should also monitor non-*enterica* Salmonella subspecies, as they could also disseminate to food production settings and eventually become an emerging public health threat.

Some of the S. enterica subsp. *enterica* strains isolated here are widespread across distant regions (i.e., serovars Senftenberg, Newport, Meleagridis, Agona, Give, Anatum, Infantis, Typhimurium) in a geographical zone that extends over 400 to 500 km. Other strains seemed more local (i.e., serovars Ohio, Bovismorbificans, Fresno), although the observed serovar diversity and representation in our study is like that reported in rivers from northwest Mexico (4, 16, 17). Therefore, it seems Salmonella contamination of SuWa in Mexico is comparable across regions, which agrees with the ubiquitous nature of the pathogen and its ability to survive under stressful conditions (3, 26, 27). In this research, we managed to isolate Salmonella from SuWa samples of varied temperature (14.5 to 32.5°C), pH (6.0 to 9.3), and turbidity (0.4 to 800 nephelometric turbidity units [NTU]). These findings are consistent with previous reports documenting the lack of association between SuWa Salmonella contamination and water physicochemical indicators (28). We did observe an association between water pH and Salmonella serovar prevalence. However, most isolates from this study (124 of 172) were collected from alkaline SuWa, causing the observed association.

Studies on fresh produce (i.e., cantaloupe, pepper, tomato, and cilantro) in the states of Coahuila, Michoacán, Guerrero, Sinaloa, and Sonora showed irrigation water was contaminated with Salmonella (29), highlighting the role of SuWa as a relevant source of Salmonella across Mexico. Other factors linked to SuWa Salmonella contamination include poor management of sewage water and wastewater from agricultural and livestock production (29, 30). These waters act as a vehicle of microbial dispersion through rain-generated flows that contaminate SuWa by runoff (3, 31). Unfortunately, wastewaters are less accessible for sampling than SuWa, and thus, they are seldom studied in conjunction with SuWa (32). Hence, further research involving both SuWa and wastewaters is required toward a more complete understanding of this phenomenon.

It is worth noting that several of our SuWa isolates belong to serovars that have been implicated in human infections previously. A recent review (2) reported that strains of serovars Typhimurium, Agona, Anatum, Enteritidis, Infantis, Muenchen, Muenster, and Ohio were major causes of human salmonellosis in Mexico between 2000 and 2017. The same review reported some of these serovars (i.e., Typhimurium, Anatum, Agona) were also frequently isolated from fruits and vegetables, beef, chicken, and pork in different studies conducted in that period (2000 to 2017). This similarity in Salmonella serovar representation across sources suggests that anthropogenic activities favor contamination of the environment by the pathogen, as well as its dissemination across food production chains, which eventually results in human exposure to the pathogen.

MLST typing and SNP phylogeny confirmed that there was a high genetic diversity and clonal dissemination of strains across the regions under study. Certain MLST profiles (i.e., ST213, ST14) are of particular interest as they have been associated with severe enteric and systemic infections (33, 34). Likewise, these clones show resistance to ceftriaxone, as well as multidrug-resistant (MDR) phenotypes (35–38). These findings are consistent with the genotypic AMR profile exhibited by our ST213 isolates. All these strains carried genes encoding a class C betalactamase (*blaCMY-2*), as well as multiple AMR alleles that confer resistance to aminoglycosides, phenicols, sulfonamides, quinolones, and tetracyclines. Likewise, close to half of serovar Senftenberg ST14 isolates had a genotypic MDR profile (*aadA*, *blaCARB-2*, *floR*, *sul1*, *qnrA1*, *tetA*, and *mph(A)*, as well as *gyrAB* mutations, *such as gyrA-*S83Y, *gyrB-*T717N) that is consistent with the penta-resistant phenotype reported for this ST (33). Although MDR genotypes were not frequently observed here (27 of 172), the clinical relevance and strong AMR profile of some strains highlight the importance of SuWa as a reservoir of AMR Salmonella and AMR genes.

The polyphyletic behavior of some serovars (i.e., Typhimurium, Newport, Senftenberg, among others) suggests that SuWa contamination arises from multiple sources. In that sense, the transmission network analysis showed that the transmission of Salmonella is a complex ecological process. However, it clearly documented how the pathogen disseminates effectively from SuWa and the environment to food sources and vice versa.

Moreover, character state reconstruction at ancestral nodes showed that the ancestors of isolates involved in human infections originated from SuWa, animals, and vegetable sources, which highlights the risk posed to public health by nonclinical isolates. However, closeness metrics indicated that every source of isolates, including human clinical cases, may also act as a direct point of transmission to other nodes. Hence, further research is needed to better assess the origins of the Salmonella circulating across ecological niches.

Phylogenetic analysis showed SuWa isolates were genetically close to clinical strains from the North America region. This relationship was further corroborated by the analysis conducted at the NCBI Pathogen Detection website. On average, close to half of our SuWa isolates (80 of 172) were less than 21 SNPs away from clinical strains belonging to the same SNP cluster, satisfying the criteria for clonality between two or more genomes (39). However, these results should be interpreted with caution since isolates from SuWa and clinical cases used in this study are not epidemiologically related. Still, their genetic proximity suggests that a considerable part of SuWa isolates are of public health significance. These findings are consistent with previous observations of high prevalence and genetic diversity of Salmonella from SuWa, as well as its genetic proximity to clinical strains in the USA and other countries (40–43), a phenomenon suggested to be favored by climate change and global warming (44–46). For instance, recent salmonellosis outbreaks in the United States have been traced back to onions, peaches, salads, papayas, and sprouts, among other vegetables originating in the United States and Mexico (47). The role of SuWa as a potential source of Salmonella contamination of these foods was not proven in these outbreak investigations. However, it should not be discarded, given its documented involvement in other salmonellosis outbreaks worldwide (7–11).

Overall, this study demonstrates the role of SuWa as a significant reservoir and a vehicle for the transmission of Salmonella in the environment and food production settings. It also highlights the need for continuous Salmonella surveillance in SuWa, as well as applying a one-health approach (i.e., by addressing wastewaters and sewage water in conjunction with SuWa, animals and foods) to facilitate the identification of the sources of Salmonella contamination, as well as control measures to contain the spread of this foodborne pathogen.

## MATERIALS AND METHODS

### Sampling scheme.

**FIG 6:**
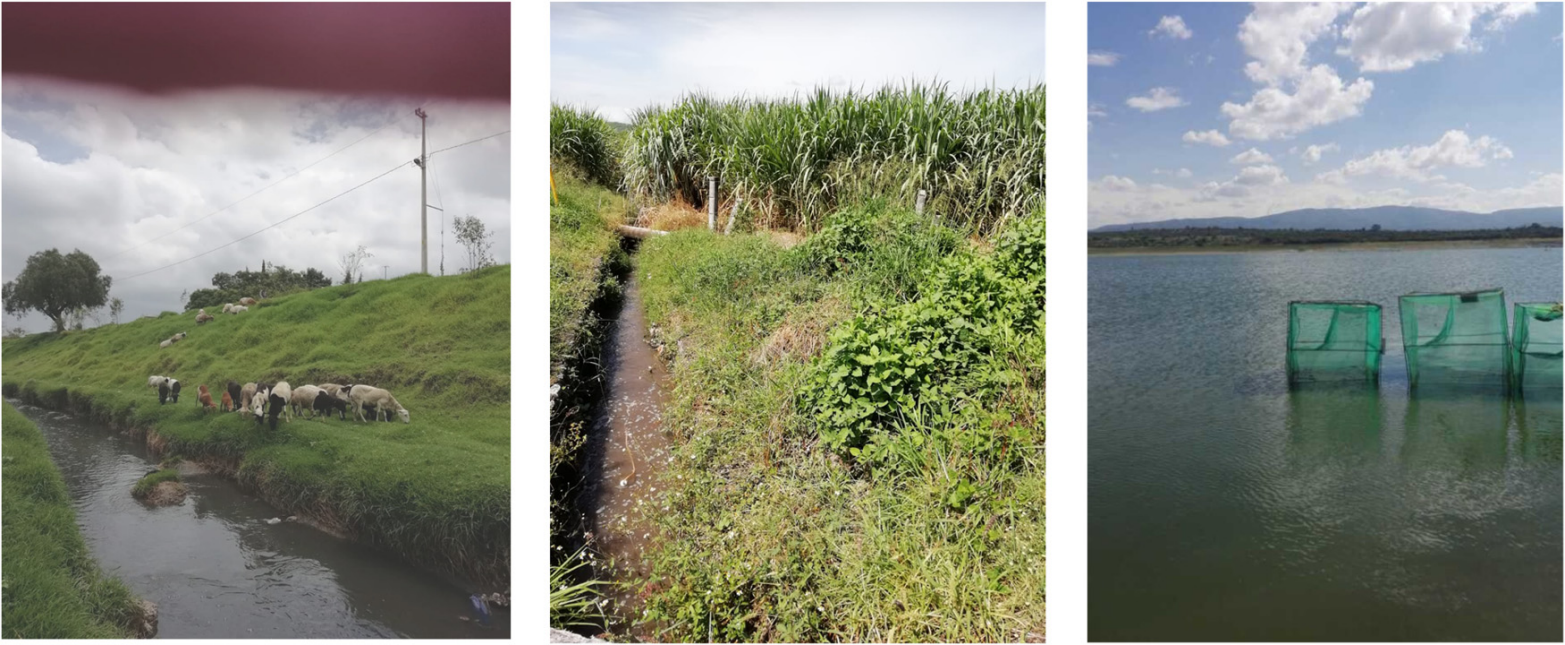
Examples of selected sampling sites nearby food production areas and their global positioning system (GPS) coordinates. (Left) River used by local sheep producers (19.29425, −98.875394). (Center) Irrigation canal used in sugarcane fields (18.78898, −99.221747). (Right) Tilapia production in a pond (20.436595, −99.36901).

Isolates used in this study (*n* = 172) originated from a genomic surveillance project in surface waters from central Mexico. This project comprises a convenience sampling scheme whereby sampling sites participating in the survey should meet the following criteria:

1 Wadeable streams with public access that can be reached by vehicle and allow performing sampling activities securely. For instance, we avoided sites located in irregular terrains, within sharp mountains or cliffs, surrounded by swamps, and where personnel security could not be guaranteed.
2 Sample sites should be in the proximity of food production areas, such as crop production, horticulture, animal husbandry, or aquaculture (Fig. 6). Sites may be different locations within a watershed (i.e., multiple locations from a river system or from a large pond/lake).
3. Surveyed sites should not be far from the laboratory to allow processing samples within 24 h after collection.

**FIG 7:**
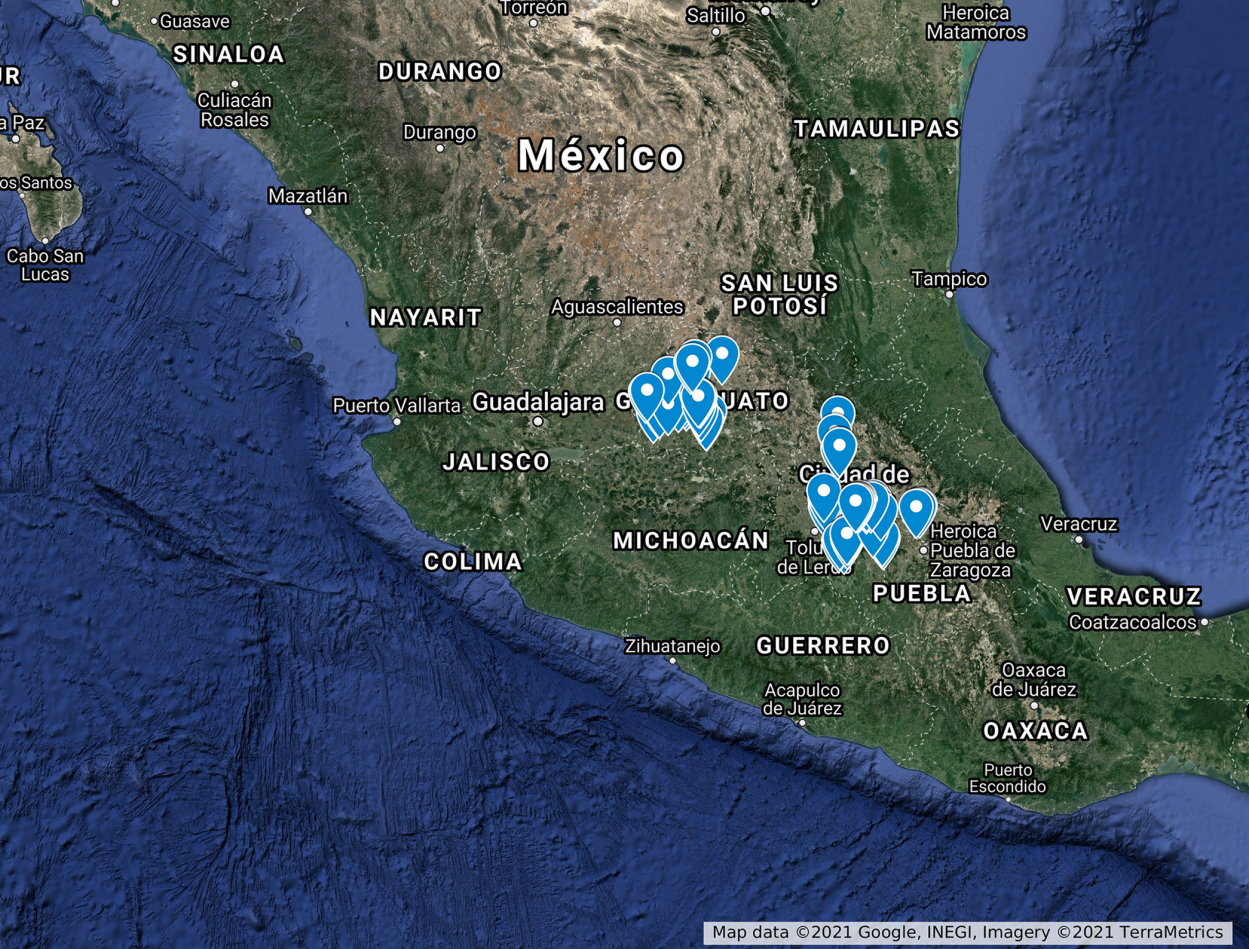
Overview of sample collection points across the six Mexican states that were included in the survey. For full details, refer to the interactive map at: https://www.google.com/maps/d/edit?mid=1dScbM7__NgBr6eoybRmPImb1qey2GcbK&usp=sharing.

According to these criteria, we took samples from rivers, dams, lakes, ponds, and irrigation canals across six Mexican states from May through October 2019. Overall, 69 different sites were visited five times during this period. In each visit, we collected one sample per site, unless the stream dried under drought conditions or landowners no longer permitted access to it. Table 2 summarizes the samples collected from May through October 2019 (*n* = 323), from which the isolates used in this study originated. Overall, we obtained 522 pure Salmonella isolates out of the 323 samples analyzed. Of these, we managed to submit 232 for WGS before the advent of the COVID-19 pandemic. After WGS, 60 duplicated isolates were discarded. Duplicated isolates originated from the same sample and belonged to the same serovar and single-nucleotide polymorphism (SNP) cluster at the NCBI Pathogen Detection website and had a SNP distance of less than 21 between them (13). After identifying duplicates, we picked the genome with the highest assembly quality: lowest number of contigs and *L*_50_ values. When contigs and *L*_50_ were comparable among duplicates, the genome with the highest depth of coverage was selected. According to these criteria, the final set of isolates included in this research was 172. Table S1 contains the accession numbers, NCBI SNP clusters, and metadata of the 232 isolates, as well as the duplicates that were discarded. Moreover, Fig. 7 provides an overview of the sampling sites surveyed in this research. An interactive Google map version is also available through the following link: https://www.google.com/maps/d/edit?mid=1dScbM7__NgBr6eoybRmPImb1qey2GcbK&usp=sharing.

**TABLE 2:** Surface water samples collected per Mexican state from May through October 2019^*a*^

Mexican state	Sites	No. of samples per water source					Total samples
		River	Dam	Lake	Pond	Irrigation canal	
Mexico City	10	5	0	0	7	26	38
Guanajuato	20	30	35	4	4	27	100
Hidalgo	8	7	25	0	0	0	32
Mexico State	12	45	0	5	5	5	60
Morelos	12	25	10	0	15	8	58
Tlaxcala	7	15	0	0	10	10	35
Overall	69	127	70	9	41	76	323

a Each site was sampled five times across 6 months unless the stream dried under drought conditions or access to it was no longer possible.

We collected SuWa samples *in situ* by the modified Moore swab (MMS) method (48), with slight modifications. The MMS was tied to a stick to prevent it from sinking to the bottom or being dragged by the stream. This practice ensured taking samples from the surface and that no sediment from the bottom was pulled into de MMS. We filtered a 10-L water volume through an MMS coupled to a peristaltic pump adjusted at 0.5 L/min of water flow. Subsequently, MMS was placed in a sterile plastic bag. The bag was then closed and transported to the laboratory in an insulated container with cooling gel pads. A full description of the MMS method used is available from protocols.io (doi: https://dx.doi.org/10.17504/protocols.io.bpw9mph6). All water samples were analyzed within 16 h after collection.

At each sampling site, we measured water physicochemical variables. For that purpose, we used a container to collect approximately 500 mL water, as close as possible to the place where the MMS was located (refer to the published protocol for full details: https://dx.doi.org/10.17504/protocols.io.bpw9mph6). We used a portable pH meter (Sper Scientific 850051, Scottsdale, AZ, USA) to measure SuWa pH and temperature. The pH meter was calibrated before each sampling session following the manufacturer’s instructions. We also measured SuWa turbidity with the aid of a portable turbidimeter HI 93703 (Hanna Instruments Mexico, Mexico City, Mexico). The results for SuWa temperature, pH, and turbidity, as well as the date and time of sampling and global positioning system (GPS) coordinates, were recorded as sample’s metadata with the aid of the Epicollect5 app (49). Some metadata were classified to assess their association with serovar and genetic diversity of Salmonella from SuWa. For that purpose, water turbidity was classified as low (20 NTU or less), intermediate (21 to 100 NTU), or high (more than >100 NTU), as previously proposed (50). Likewise, water pH was classified as acidic (pH less than 7.0), neutral (7.0), or alkaline (pH greater than 7.0), while water temperature was classified as low (less than 20°C) or high (more than 20°C).

### Salmonella isolation and confirmation.

For Salmonella isolation and confirmation, we used a modified version of the US Food and Drug Administration (FDA) Bacteriological Analytical Manual methodology (51). Briefly, preenrichment (36°C/18 h) of MMS filters was conducted in 200 mL of modified buffered peptone water (mBPW). From the preenriched sample, a 0.1-mL aliquot was transferred into an assay tube containing 10 mL of Rappaport Vassiliadis Soya (RVS) broth. Likewise, we took 1.0 mL of the preenriched sample and transferred it into an assay tube containing 9-mL tetrathionate (TT) broth. After vortexing for 3 to 5 s, RVS and TT broths were incubated at 43 and 42°C, respectively, for 18 to 24 h. After incubation, a 3-mm (10 μl) complete loop of RVS and TT broths was plated, in duplicate, onto xylose-lysine-Tergitol (XLT-4) and CHROMagar Salmonella selective agar and incubated at 35°C for 24 h.

After incubation, we picked two or three isolated colonies showing the typical Salmonella morphology from each selective agar plate (maximum 10 colonies/sample) to conduct molecular identity confirmation. We then used a PCR test targeting the *invA* gene (28- bp fragment), as previously described (52). DNA was extracted using the Ez-10 spin column bacterial genomic DNA miniprep kit (BioBasic, Inc., Canada), following the instructions of the supplier, from pure strains, previously refreshed in tryptic soy broth for 18 to 24 h. Forward (CGCCATGGTATGGATTTGTC) and reverse (GTGGTAAAGCTCATCAAGCG) primers were used in PCR with a total volume of 10 μl, employing the Firepol Master Mix reagents (Solis BioDyne, Estonia) with the following final concentrations: 5 μl of Firepol Master Mix, 0.2 μl of each dNTP, and 2.1 μl of nuclease-free water. The thermocycling conditions were the following: 94°C/3 min of initial denaturation; 35 denaturation, annealing, and extension cycles (95°C/45 s, 62°C/30 s, 72°C/45 s, respectively), and a final extension at 72°C/2 min. The PCR-amplified products were run in 2% agarose gel electrophoresis. The gels were run in a Tris/borate/EDTA buffer (TBE 1×) at 80 V for 50 min using SYBR Safe DNA gel stain (Invitrogen, USA) to reveal the DNA fragments. The visualization and digitization of images were performed in a Gel Logic 2200 imaging system (Kodak, USA) with Care Stream software (Carestream Health, Inc., USA). In each run, we included a positive-control strain from our laboratory: S. enterica subsp. *enterica* ser. Typhimurium, previously subjected to whole-genome sequencing (NCBI Biosample SAMN15872722). A detailed description of Salmonella isolation and PCR confirmation methods used here is available from protocols.io (doi: https://dx.doi.org/10.17504/protocols.io.bpybmpsn).

For long-term preservation, pure Salmonella isolates were stored at −80°C in vials containing brain heart infusion broth and 30% glycerol. From the glycerol stock, bacteria were recovered and streaked onto slants of semisolid tripticase soy agar (TSA) and shipped to the molecular biology laboratory of the Center for Food Safety and Applied Nutrition (FDA, MD, USA) for WGS. The input material for WGS was a cell pellet of a 1-mL fresh bacterial culture grown in Luria Bertani broth at 37°C overnight.

### Whole-genome sequencing.

Genomic DNA was extracted with the aid of the Qiagen QIAsymphony system using QIAsymphony DSP DNA kit. Subsequently, it was quantitated using Qubit fluorometric quantitation (Life Technologies), according to the manufacturer’s instructions. Next, we prepared DNA libraries from 1 ng of genomic DNA using the Nextera XT DNA sample preparation kit, version 2 (Illumina). DNA libraries were then sequenced on the Illumina MiSeq system (paired-end 2 × 250-bp reads). Raw reads were deposited at NCBI, and genome assemblies are publicly available through the accession numbers reported in Fig. 2.

### Data quality control and genome assembly.

The quality of raw reads was assessed with FastQC (53), and we used Trimmomatic version 0.39 (54) to filter Illumina adaptors and reads with a Phred quality score (Q) below 30. Trimmed sequences were subjected to *de novo* assembly using SPAdes version 3.13.1 (55), while the quality of the assemblies was assessed with QUAST version 5.02 (56). A summary of genome assembly results is also provided in Table S1.

### *In silico* serovar prediction and genotypic antimicrobial resistance profiling.

Salmonella serovars were predicted with the aid of SISTR software, version 1.1.1 (57). For ambiguous predictions, we chose the serovar predicted by SISTR through cgMLST. In all cases, the cgMLST prediction was corroborated as isolates on the same SNP cluster at NCBI Pathogen Detection website belonged to the same serovar. Moreover, AMR genes and point mutations associated with AMR were predicted with AMRFinderPlus version 3.10.1 (58). Both analyses were conducted with the assembled genomes. Isolates with AMR genes against three or more antimicrobial classes were classified as genotypically multidrug resistant (MDR) (59).

### MLST, phylogenetic analysis, and genotypic AMR typing.

The genetic relatedness among the newly sequenced 172 Salmonella isolates was evaluated through MLST typing and SNP phylogeny. MLST typing was conducted at the Center for Genomic Epidemiology website (http://genomicepidemiology.org/) using assemble genomes in MLST version 2.0 (60). For SNP-based phylogeny, we used Salmonella Typhimurium LT2 as a reference (accession no. NP_460230.1). The SNPs were located, filtered, and validated using CSI Phylogeny 1.4, with default values (61). The resulting alignment was analyzed with RAxML, version 8.0, to generate a maximum likelihood (ML) tree, under the GTR + Γ model of nucleotide evolution at the CIPRES Science Gateway server, version 3.3 (62). The analysis was run with default values, using the fast bootstrap algorithm with 100 iterations. The resulting tree was displayed in FigTree version 1.4.3 and edited using iTOL version 6 (63). AMR genotypes were mapped onto the tree to explore whether there was an association between specific clades and AMR profiles.

### Salmonella transmission dynamics.

To study the pathogen transmission dynamics within Mexico, we constructed a transmission network using StrainHub, version 0.2.0 (64). For that purpose, we picked one representative isolate from each subclade of isolates belonging to the same Salmonella serovar in the previously generated ML tree through SNP analysis. For isolates showing polyphyletic behavior, we picked at least one representative strain from each subclade where these serovars were present. These data were complemented with public isolates from Mexico belonging to the same serovars (if available) and isolated from human clinical cases, animals, vegetables, surface waters, and other environmental sources in 2002 through 2019. Overall, the analysis included 199 isolates of 38 different serovars from 15 different locations across Mexico (Fig. 4). To run StrainHub, we first generated an ML tree, as described previously (Fig. S1), and used it as input along with a csv file containing isolate metadata. Then, the software constructed the network by mapping the metadata onto the tree and performing a parsimony ancestry reconstruction step to create links between the associated metadata and generate the network transmission metrics. Each metric has an epidemiological meaning, as follows (64):

1. Source/hub ratio: how important each node is as the source of the disease, ignoring centrality within the network;
2. Betweenness centrality: how important a node is, like the shortest path/intermediary connecting other nodes within the transmission network;
3. Closeness centrality: how important a node is given its distance within the transmission network to other nodes; and
4. Degree centrality: how vital a node is within the transmission network given the number of times that the pathogen/disease emerges from (outdegree) or to (indegree) that point.

In the transmission network scheme, the arrows reflect the directionality of transition between states, while the thickness of lines and arrows represents the frequency of transitions (thicker arrows reflect more transitions) (64).

To further corroborate transmission network analysis, we used Mesquite software, version 3.70, to reconstruct the character states at ancestral nodes, using a parsimony unordered model on the same phylogenetic tree (65). Through this analysis, we obtained the summary of character state changes across the tree, as well as a color-coded tree showing the character state transitions.

### Genetic relatedness of isolates from SuWa and clinical strains from North America.

To assess the public health significance of strains circulating in SuWa, we constructed another ML tree with representative isolates from our study, as well as public isolates involved in human infections across Mexico, the USA, and Canada in 2005 through 2020. To select the isolates, we picked representative SuWa strains from each serovar that has clinical counterparts from any of the above-mentioned countries at NCBI. Overall, the data set was composed of 33 Salmonella isolates from 12 different serovars (Fig. 5). The ML tree was generated as previously described here. To obtain a better picture of the potential clinical relevance of SuWa isolates, we also checked the NCBI SNP clusters reported at the NCBI pathogen detection website for each of our experimental isolates. In that way, we managed to identify the proportion of our SuWa isolates that are closely related to clinical strains in the public database.
