# Supplementary material for: Genomic Epidemiology of Salmonella enterica Circulating in Surface Waters Used in Agriculture and Aquaculture in Central Mexico
Source: Appl Environ Microbiol. 2022 Mar 8;88(5):e02149-21. doi: 10.1128/aem.02149-21 (PMC8904062; doi:10.1128/aem.02149-21)
Supplement: Supplemental file 1 — Fig. S1 and S2. Download aem.02149-21-s0001.pdf, PDF file, 6.7 MB [file aem.02149-21-s0001.pdf]

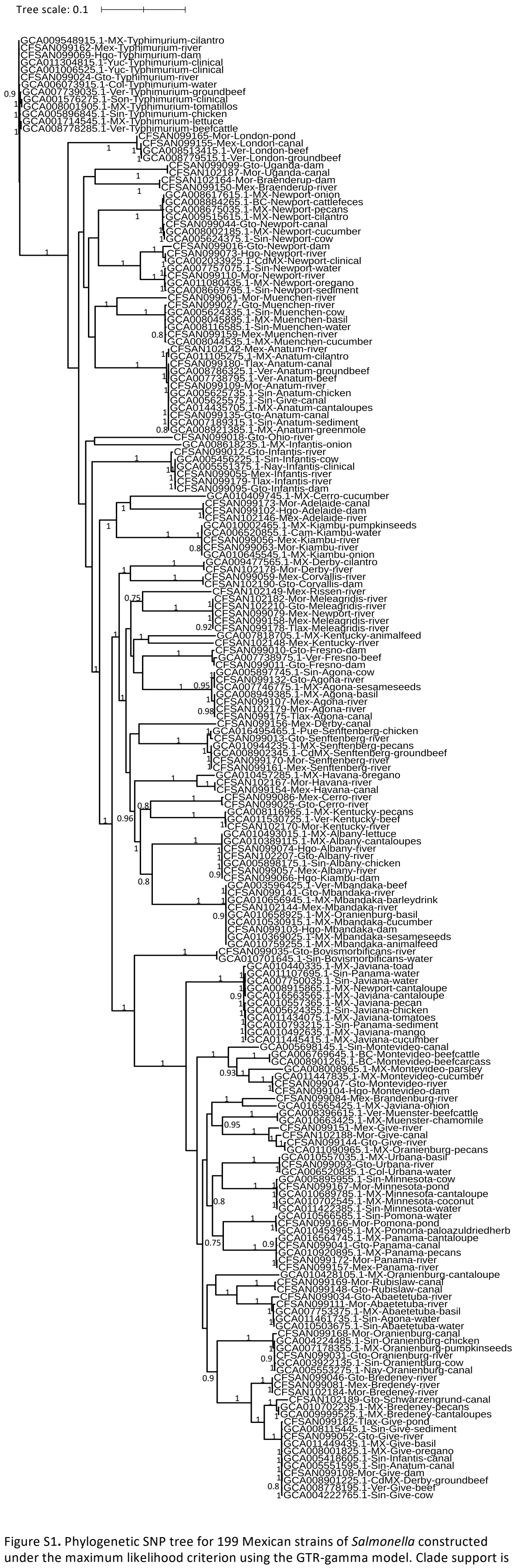

Figure S1**.** Phylogenetic SNP tree for 199 Mexican strains of *Salmonella* constructed under the maximum likelihood criterion using the GTR-gamma model. Clade support is indicated in the branches as bootstrap values, unless <0.7. Gto: Guanajuato; MX: Mexico; Mex: State of Mexico; Mor: Morelos; Hgo: Hidalgo; CdMX: Mexico City; Tlax: Tlaxcala; Yuc: Yucatan; Col: Colima; Ver: Veracruz; Sin: Sinaloa; Son: Sonora; Nay: Nayarit; BC: Baja California; Pue: Puebla; Cam: Campeche.

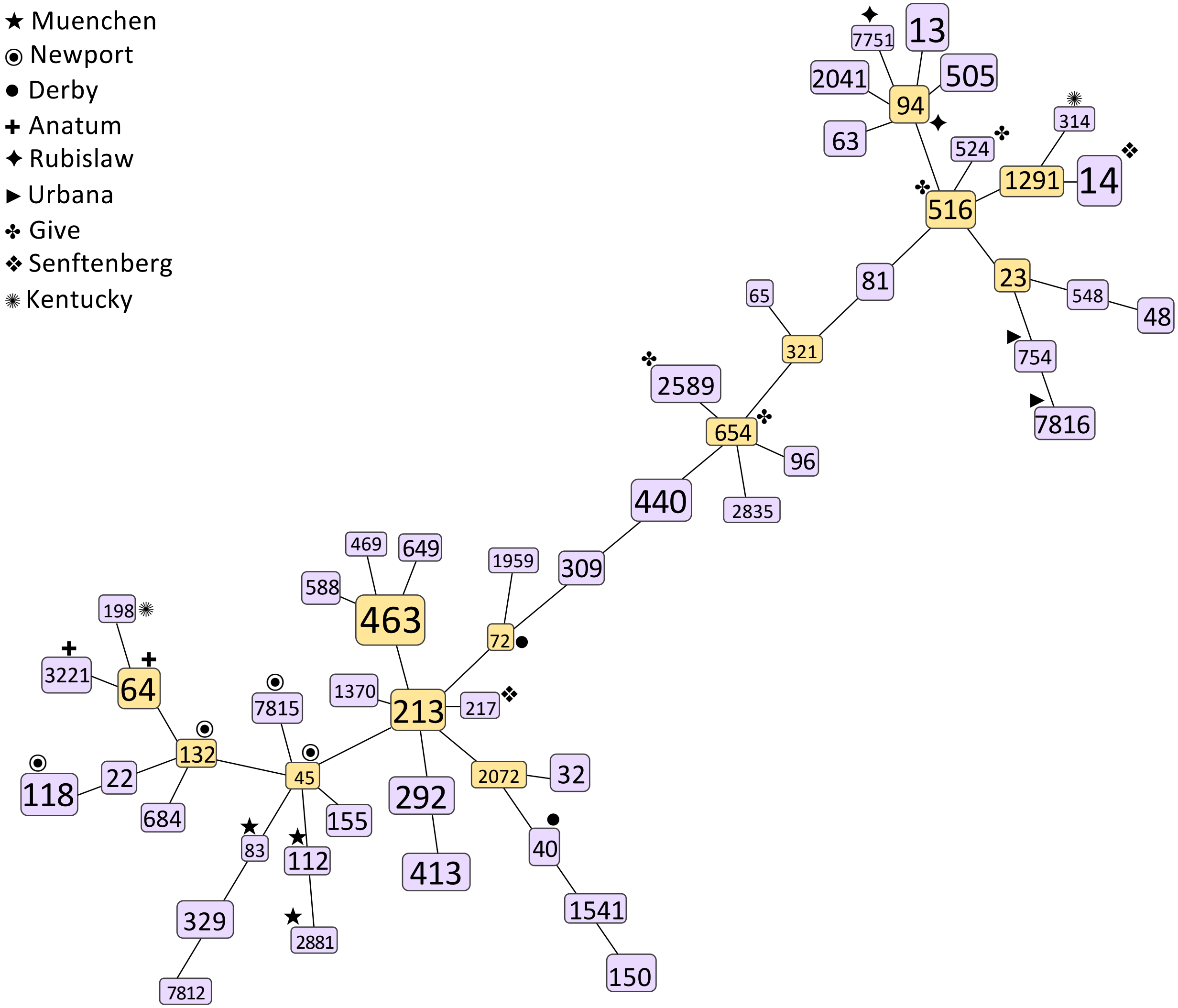

Figure S2. Minimal spanning tree of MLST data on 172 isolates of *Salmonella* spp. Each number corresponds to one of fifty-six STs, whose size is proportional to the number of isolates. The topological arrangement within the MSTree is proportional to its graphic algorithm, which uses an iterative network approach to identify sequential links of increasing genetic distance, beginning with the central STs. The STs in yellow correspond to founders. Symbols above some STs correspond to the STs relational with the polyphyletic serotypes Muenchen, Newport, Derby, Anatum, Rubislaw, Urbana, Give, Senftenberg and Kentucky.
